# Construction of Efficient 3D Gas Evolution Electrocatalyst for Hydrogen Evolution: Porous FeP Nanowire Arrays on Graphene Sheets

**DOI:** 10.1002/advs.201500120

**Published:** 2015-06-03

**Authors:** Ya Yan, Larissa Thia, Bao Yu Xia, Xiaoming Ge, Zhaolin Liu, Adrian Fisher, Xin Wang

**Affiliations:** ^1^ School of Chemical and Biomedical EngineeringNanyang Technological University50 Nanyang AvenueSingapore639798Singapore; ^2^ Institute of Materials Research and Engineering (IMRE)Agency of Science, Technology, and Research (A*STAR)3 Research LinkSingapore117602Singapore; ^3^ Department of Chemical Engineering and BiotechnologyUniversity of CambridgeNew Museums Site, Pembroke StreetCambridgeCB2 3RAUK

**Keywords:** hierarchical nanocomposites, hydrogen evolution reaction, porous FeP nanowire arrays, pseudomorphic transformation

## Abstract

**A novel 3D hierarchical nanocomposite of vertically aligned porous FeP nano­wires on reduced graphene oxide** is prepared as a demonstration of constructing an efficient hydrogen evolution catalyst. Extension of this nanostructuring strategy to other functional nanocomposites by combining different dimensional nanomaterials is attractive.

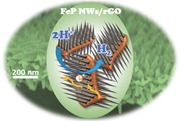

Hydrogen is being regarded as a clean energy carrier as it is environmentally friendly and possesses high energy density. The development of low cost and efficient electrocatalysts for hydrogen evolution reaction (HER) is critical if large scale production of hydrogen is to be realized via water electrosplitting. In the past decade, transition metal chalcogenides,^1^ nitrides,^2^ and carbides^3^ have been extensively studied for this purpose. Recently, transition metal phosphides (TMPs) have also attracted much interest as they are active toward HER in electrolytes with a wide range of pH.^4^ Iron phosphide (FeP), a metal‐rich TMP, exhibits physical and chemical properties similar to carbides and nitrides. It is a good conductor of heat and electricity and also has high thermal and chemical stability.[[qv: 4k]] In addition, unlike transition metal chalcogenides, e.g., MoS_2_, FeP does not form layered structures. Hence, FeP potentially allows better access to active corner and edge sites on the crystallite surface, making it uniquely advantageous as an electrocatalytic material.^5^ Lately, renewed studies have demonstrated the encouraging HER activity of FeP based catalysts.^6^ However, further advances in FeP HER catalysts, are hindered by their unfavorable H_2_ release capability and limited cycling stability,^7^ which may be due to the low porosity of the material or the high‐adhesion between the surface of the catalytic electrode and the as‐formed H_2_ bubbles.

Constructing composite materials is a promising strategy to help overcome the drawbacks of single material usage whilst simultaneously deriving materials having both high activity and robust structures.^10^ Composite materials also enable the combination of the unique properties of individual nanostructures and harness any potential synergistic effects. As such, by function‐oriented selection of nanocomponents, well‐ordered nanocomposites can be expected to exhibit high HER performance. Recent strategies for designing highly efficient HER catalysts^9^ already suggest that hierarchical nanostructures, assembled from low‐dimensional building blocks such as 1D nanorods or nanowires (NWs), are promising structures for the hydrogen evolution process.[[qv: 7a,b]],^8^, ^10^

Compared to randomly connected nanowires or aggregated nanowire bundles, well‐ordered nanowires or nanorod arrays can reduce the ionic diffusion path, facilitate ionic motion to the inner part of electrode, and improve utilization of electrode materials. In particular, when functioning as a gas evolution electrode, such hierarchical arrays favor rapid gas bubble release. However, nanostructure arrays have mostly been produced on bulky substrates,[[qv: 7a,b]],[[qv: 8c]]which makes it difficult to adjust catalyst loading at the device level. There is no report on the construction of hierarchical materials by combining 1D FeP nanowires with low‐molecular‐weight 2D nanosheets. Thus, in order to fully reap the advantages of using well‐ordered nanostructure arrays, we selected graphene nanosheets as substrates to synthesize a novel 3D hierarchical nanocomposite consisting of porous FeP nanowire arrays grown vertically on 2D reduced graphene oxide (rGO) (denoted as FeP NWs/rGO). We first constructed vertically oriented FeO(OH) nanowires supported on graphene oxide (GO) (namely FeO(OH) NWs/GO) which served as the precursor. 3D FeP NWs/rGO pseudomorphism was then successfully achieved via phosphorization transformation of the FeO(OH) NWs/GO precursor. The 3D FeP NWs/rGO electrode exhibited high and stable activity towards HER. As expected, HER performance of 3D FeP NWs/rGO also exceeded that of their single component counterparts.

The first and key step of this synthesis involves hierarchical growth of the nanowire‐structured iron oxide precursors onto the GO sheets via an interesting quasi‐emulsion‐assisted hydrothermal method. Hydrolysis of iron salts in aqueous solution has previously been successfully employed for the preparation of iron oxide nanowire arrays on various bulky substrates, such as Ti plate or carbon cloth (CC).[[qv: 7a,b]],[[qv: 8c]]In our study, we expected that iron oxide nanowire arrays produced would similarly be vertically aligned on the 2D GO nanosheets (see the Supporting Information for details). As illustrated in **Scheme** **1**, FeSO_4_·7H_2_O was first dissociated in a mixture containing deionized water (DI) water and glycerol. It was then converted to ferric ions which were attached to the neutral glycerol ligands during the hydrolysis process^11^ When GO was employed as a support and dispersed into the solvents, the polar glycerol solute uniformly attached onto the surface of the GO sheets to form a glycerol quasi‐emulsion coated film.^12^ Most α‐FeO(OH) active nucleation sites are then generated on the surface of the glycerol coated GO nanosheets by heterogeneous nucleation at the beginning of the hydrolysis process. These active sites minimized the interfacial energy barrier between the solid surface and bulk solution, and presence of these active sites was beneficial to the subsequent growth of α‐FeO(OH) on the solid substrates.^13^ During the prolonged hydrothermal treatment, α‐FeO(OH) nanowires further grew from the initial nuclei, eventually producing aligned α‐FeO(OH) nanowires on the GO nanosheets while GO itself was partially reduced. At the pseudomorphism stage, FeP NWs/rGO nanocomposites are produced through pseudomorphoic transformation of the as‐formed FeO(OH) NWs/rGO via low‐temperature phosphorization in the presence of sodium hypophosphite (NaH_2_PO_2_). Thermal decomposition of NaH_2_PO_2_ enables FeO(OH) NWs to be transformed in situ into FeP. Partially reduced GO is also further reduced to rGO by PH_3_ released from NaH_2_PO_2_. Direct growth of FeP on rGO substrates ensures a low contact resistance between the substrate and the electrocatalyst.

**Scheme 1:**
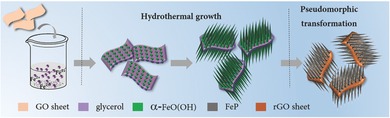
Schematic illustration of the formation process of hierarchical FeP NWs/rGO nanocomposite.

Freshly synthesized FeO(OH) NWs/rGO are received in a hydrogel format (inset of **Figure** **1**A). It has a well‐defined, interconnected 3D porous network as imaged from its freeze‐dried samples (Figure 1A). Solution phase synthesis of the α‐FeO(OH) NWs on GO sheets tend to result in a surface topography that is dominated by NW products. As expected, the hierarchical FeO(OH) nanowires grew evenly on the GO sheets; they are also highly oriented and uniformly aligned on the surface of GO sheets with little bending (GO sheet is indicated by red dash line, Figure 1B). These nanowires show a fairly consistent diameter of about 30 nm with varying lengths of 200–300 nm (inset of Figure 1B). Intimate integration of the α‐FeO(OH) nanowires with the GO surface could impart enhanced mechanical stability. Upon thermal phosphorization, α‐FeO(OH) NWs/rGO is fully converted to FeP NWs/rGO with retention of their 3D porous network (Figure 1C). Upon a closer examination (Figure 1D), the intact 1D morphology can be easily observed, albeit with an apparent increase in surface roughness, likely due to the gradual chemical conversion of FeO(OH) to FeP.[[qv: 7a]],^14^ Compared to FeO(OH) NWs, FeP NWs have similar diameters but decreased lengths in the range of 100–200 nm with a lower aspect ratio (inset of **Figure** **2**D). Energy‐dispersive X‐ray (EDX) elemental mapping images (Figure 2E) of the composite confirm the homogeneous distribution of Fe and P elements throughout the rGO sheets. Finally, X‐ray diffraction (XRD) analysis of the as‐prepared α‐FeO(OH) NWs/rGO precursors and FeP NWs/rGO confirms the overall phase purity and successful conversion of α‐FeO(OH) (JCPDS no. 29‐0713) to FeP (JCPDS no. 65‐2595) (Figure 1F). These results correspond well with that expected for nanocrystalline MnP‐type FeP. Note that, in the XRD pattern for α‐FeO(OH) NWs/rGO, diffraction from rGO is not visible due to heavy product coverage on the substrate. This could also indicate that rGO experienced almost no aggregation and was fully used as the substrate for FeO(OH) NWs nanowires.

**Figure 1:**
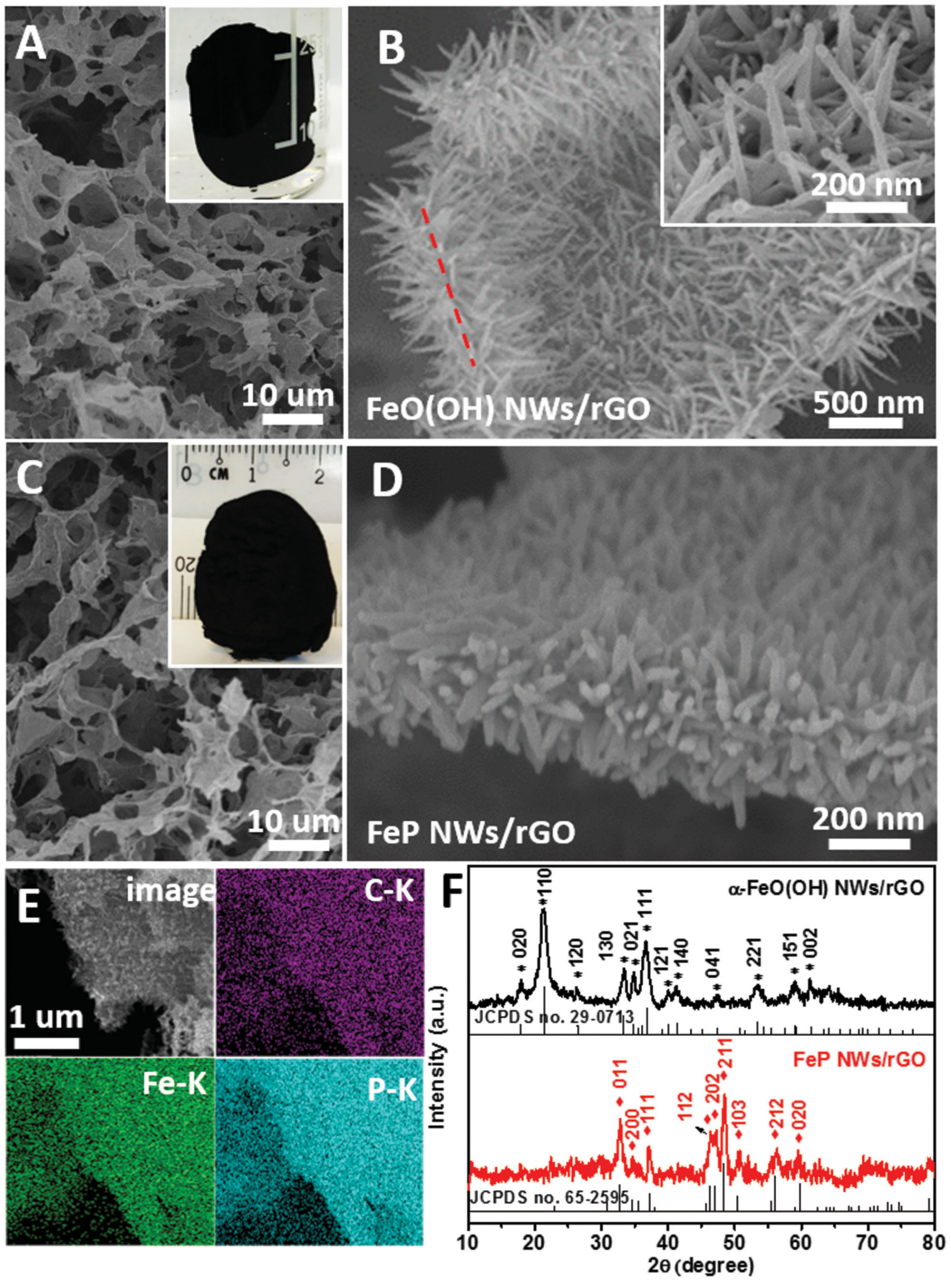
A,B) Low‐ and high‐magnification SEM images of the FeO(OH) NWs/rGO precursor, inset of A is the photograph of the precursor obtained after hydrothermal reaction and inset of B is enlarged SEM image of the vertically aligned FeO(OH) NWs. C,D) Low‐ and high‐magnification SEM images of FeP NWs/rGO, inset of C is the photograph of the phosphorized product. E) The SEM image and the corresponding EDX elemental mapping of C, Fe, and P for FeP NWs/rGO composite. F) XRD patterns of the as‐prepared FeO(OH) NWs/rGO precursor and FeP NWs/rGO.

**Figure 2:**
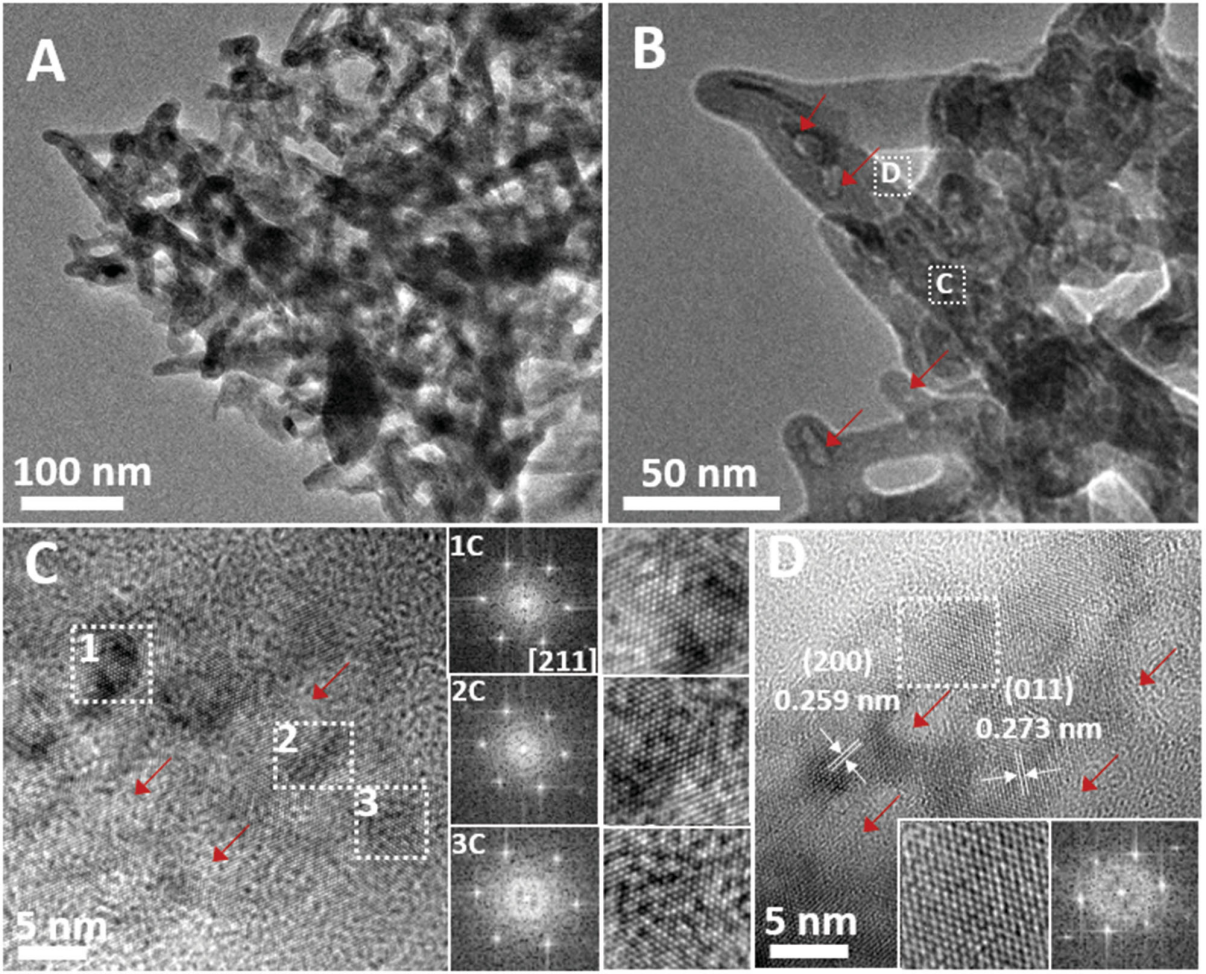
A,B) TEM image of FeP NWs/rGO. C,D) HRTEM image taken from the area as marked in (B) and the corresponding FFT patterns with inverse FFT images for selected areas (inset) (the red arrows index the pores among the nanowires).

Figure 2A shows transmission electron microscopy (TEM) images of FeP NWs/rGO. Complete coverage of FeP nanowires on the rGO sheets and preservation of the 1D morphology (Figure S2, Supporting Information) after phosphorization is again observed. Upon closer examination (Figure 2B), the porous structure among the nanowires can be easily observed (pore indicated by the red arrows). High‐resolution TEM (HRTEM) images of the selected areas in Figure 2B reveals that the porous nanowires are single crystalline and faceted (Figure 2C,D). Fast Fourier transform (FFT) patterns inset are indexed to the [211] zone axis of FeP NWs. The lattice fringes observed by HRTEM were 2.7 and 2.6 Å, which corresponds well to the (011) and (200) planes of MnP‐type FeP, respectively (Figures 2D and S1, Supporting Information) and is consistent with bulk XRD data. Such an interesting structure renders the sample a high Brunauer–Emmett–Teller (BET) area and pore volume of 95 m^2^ g^−1^ and 0.24 cm^3^ g^−1^, respectively. The sample also exhibits a narrow pore size distribution with a significantly higher pore volume (0.022 cm^3^ g^−1^ Å^−1^) at the main pore diameter of 40 Å (N_2_ adsorption desorption isotherm of the sample is provided in Figure S2, Supporting Information). This result is consistent with the TEM observation that uniform pore structure in FeP NWs/rGO was successfully formed.

Further evidence for the formation of the FeP NWs/rGO composites was obtained from X‐ray photoelectron spectroscopy (XPS) analysis. The XPS survey spectrum (**Figure** **3**A) shows that the sample surface consists of C, O, P, and Fe elements, the element O could come from residual oxygen‐containing functionalities in the rGO and surface oxidation of FeP NWs due to air contact. The mole ratio of Fe:P is 1:1.12, which is close to that of FeP. The high‐resolution XPS spectrum of the C1s (Figure 2B) proves the reduction of GO to rGO as evidenced by considerable deoxygenation of GO through the successively solvothermal and thermal phosphorization,^15^ which is very important for ensuring high conductivity of FeP NWs/rGO. The Fe 2p spectrum (Figure 2C) shows two distinct peaks located at 708.9 and 722.1 eV which correspond to the binding energy (BE) of Fe 2p_3/2_ and Fe 2p_1/2_ in FeP.^16^ The P 2p spectrum (Figure 2D) shows two peaks at 129.3 and 130.2 eV corresponding to the BE of P 2p_1/2_ and P 2p_3/2_, respectively. This doublet (129.3/130.2 eV) can be assigned to P bonding to Fe.^17^ While the high binding energy peak at 134.3 eV could be due to surface PO_4_^3−^ species produced when the catalyst was exposed to air.^18^ In addition, the XPS of α‐FeO(OH) NWs/rGO are shown in Figure S3 (Supporting Information) for reference.

**Figure 3:**
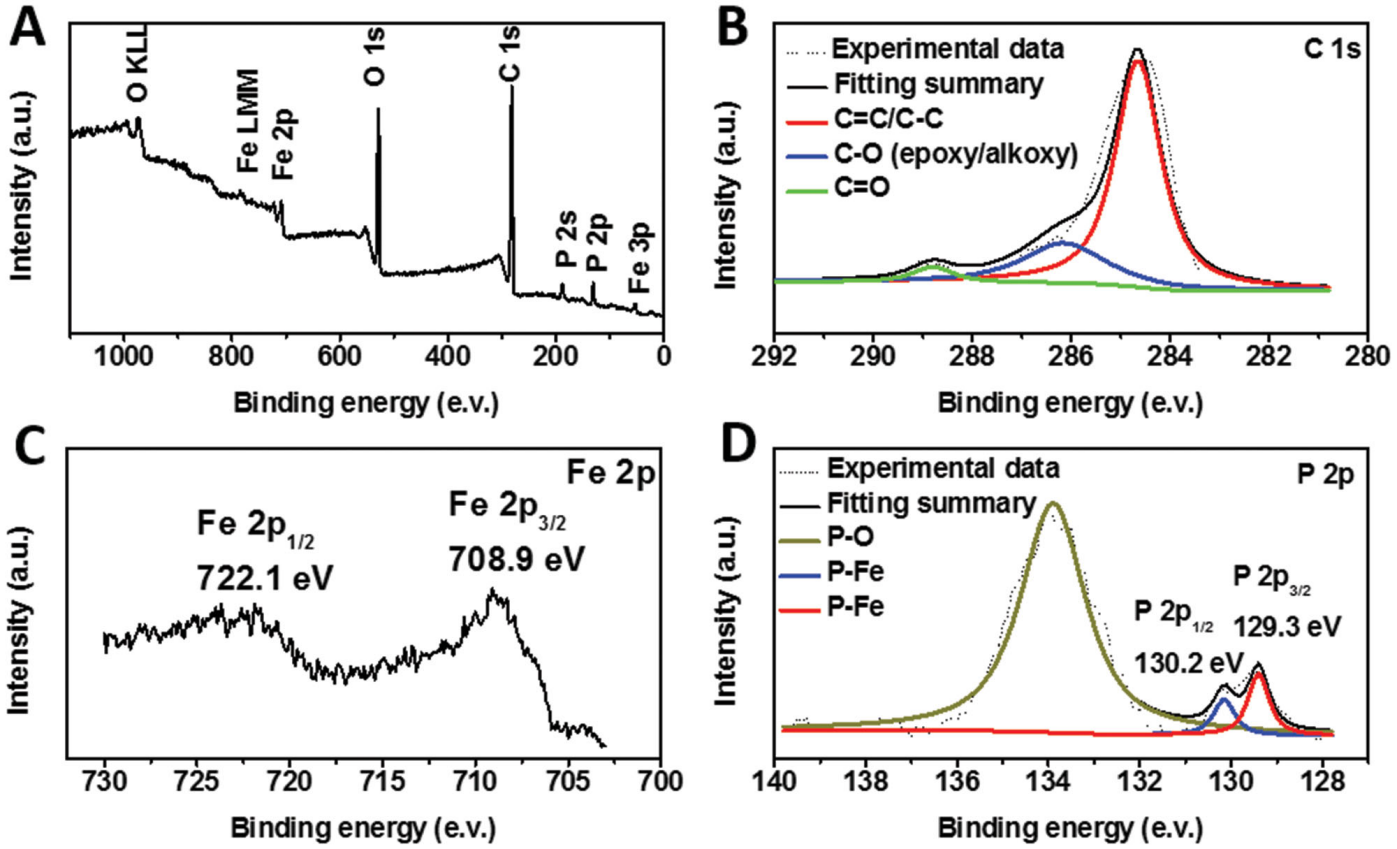
A) XPS survey scan and high‐solution spectra of B) C 1s, C) Fe 2p, and D) P 2p for FeP NWs/rGO.

To understand how the morphology of the products is affected by the presence of GO, we repeated the above procedure in the absence of GO. This resulted in the formation of spherical particles (≈1.5 μm in diameter) composed of densely packed nanowire‐like FeO(OH) subunits (Figure S4A, Supporting Information). The phosphorized product maintained a similar morphology to that of its precursor, denoted as FeP NWs assembly (shown in Figure S4B,C, Supporting Information), this observation highlights the structural guiding role of rGO in the formation of 3D FeP NWs on rGO. Nitrogen adsorption/desorption experiments (Figure S4D, Supporting Information) show that the obtained FeP NWs assemblies exhibit a BET area and pore volume of only 24.4 m^2^ g^−1^ and 0.08 cm^3^ g^−1^, respectively. These values are much lower than the corresponding values (95 m^2^ g^−1^ and 0.24 cm^3^ g^−1^) estimated for FeP NWs/rGO. It is likely that GO nanosheets function as an effective growth support and their employment gives rise to an intimate interfacial interaction between itself and the FeP NWs; These FeP nanowires, in turn, could help prevent the restacking of rGO sheets and create large voids between rGO layers. As such, the hierarchical FeP nanowires arrangement on rGO sheets produced more open channels and allowed the inner surfaces of the nanocomposite to be effectively utilized. To understand how solvent affects the synthesis process, another control experiment was conducted using only DI water. In this case, large amounts of randomly connected FeO(OH) nanowires were observed on the rGO sheets (Figure S5A, Supporting Information). In the absence of GO sheets, the obtained FeO(OH) products give rise to the bundle‐like structures (Figure S5B, Supporting Information). These results demonstrate the significant role of glycerol as oil‐in‐water quasi‐emulsion for the hierarchical growth of FeO(OH) nanowires on rGO surface. Finally, it is worth noting that the freeze dying step also plays an important role for retaining the 3D morphology of the final products (see Figure S5C, Supporting Information)

Next, we compared the electrocatalytic properties of the as‐obtained FeP NWs/rGO with FeP nanoparticles deposited on rGO (denoted as FeP NPs/rGO, see Figure S6, Supporting Information) and FeP NWs assemblies. Electrocatalysis was carried out on a glassy carbon working electrode using standard rotating disk electrode apparatus. All measurements of HER activity were performed in 0.5 m H_2_SO_4_ (*aq*) electrolyte continuously purged with H_2_ (*g*) and were corrected for background current and *iR* losses (see Figure S7, Supporting Information). **Figure** **4**A shows the polarization curves of representative samples of different FeP morphologies, along with that of a Pt wire standard and bare rGO. It can be seen that catalyst morphology clearly plays a key role in the performance as high current densities (e.g., >100 mA cm^−2^) were achievable only with the FeP NWs/rGO sample. Figure 4B highlights this comparison over the range of current densities below 10 mA cm^−2^. Specifically, to achieve current densities of 10 mA cm^−2^, an overpotential (*η*) of just 107 mV is required for the FeP NWs/rGO hybrid whereas FeP NPs/rGO and FeP NWs assemblies showed a current density of only 3.9 and 1.5 mA cm^−2^, respectively, under the same overpotential. Moreover, the FeP NWs/rGO nanocomposite shows an onset potential as low as 30 mV versus HER (determined from the semilog plot as shown in Figure S8, Supporting Information), which is smaller than that of FeP NPs/rGO and FeP NWs assemblies. Its performance also compares favorably with that of reported FeP electrode materials and other earth‐abundant HER electrocatalysts (see Table S1, Supporting Information). This suggests that the construction of well‐oriented nanoporous structures is of importance for optimizing the electrochemical performance of HER. Compared to the performance of FeP NPs/rGO, the good HER activity demonstrated by this 3D architecture could be attributed to: improved mass transfer and the increased number of exposed active sites.

**Figure 4:**
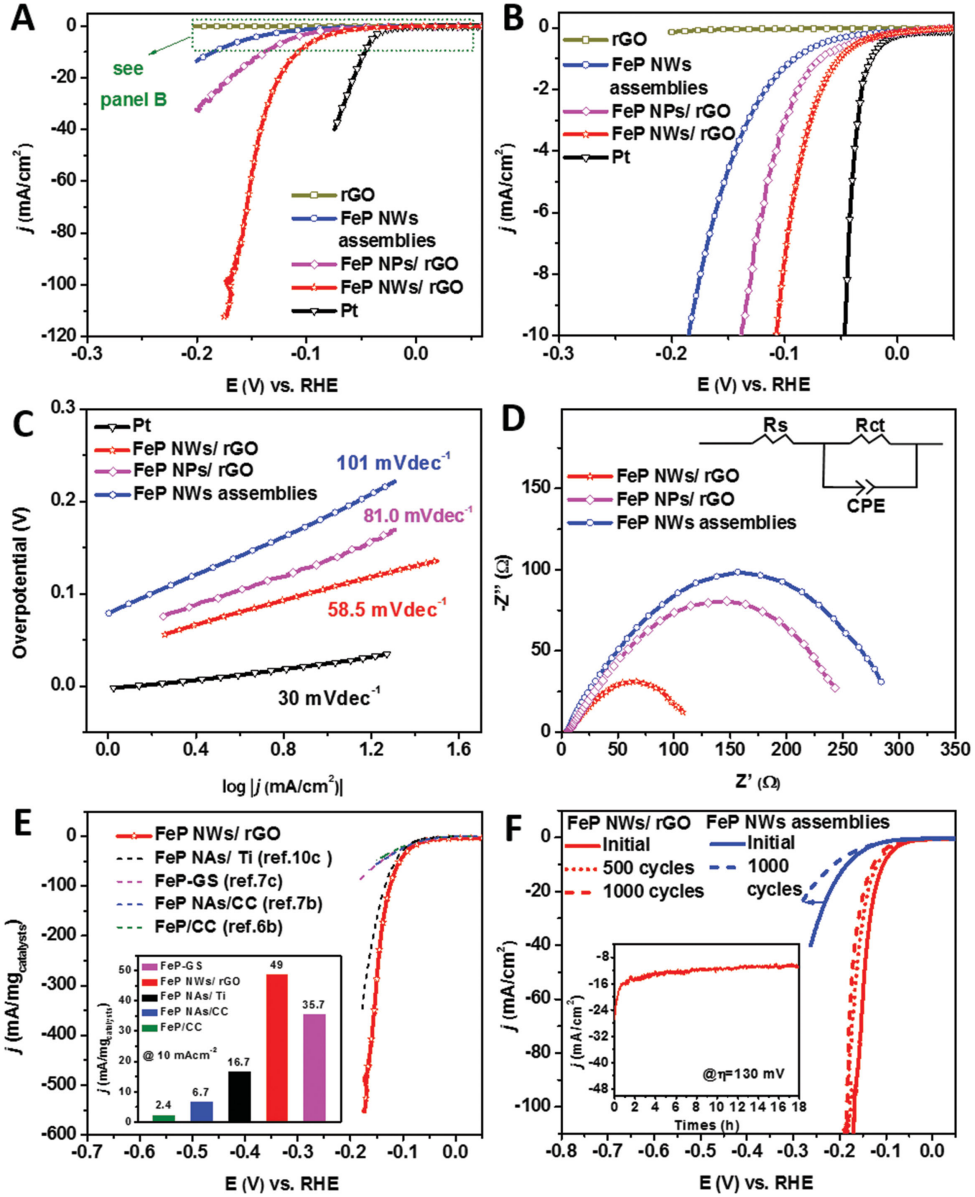
A,B) Polarization curves (B shows polarization curves with current density below 10 mA cm^−2^). C) The corresponding Tafel plots. D) Electrochemical impedance spectra at −0.09 V versus RHE (inset is the electrical equivalent circuit models for fitting the EIS response of HER on the samples). E) Mass activity for FeP NWs/rGO and these referred catalysts, which are given as current densities (*j*) normalized in reference to the loading amount of the catalysts (inset: the *j* calculated at geographic current density of 10 mA cm^−2^). F) Polarization curves for FeP NWs/rGO and FeP NWs assemblies in 0.5 m H_2_SO_4_ initially and after 500 and 1000 cycles at a scan rate of 50 mV s^−1^ (inset: plot of current density vs time for FeP NWs/rGO electrode under static *η* = 130 mV for 18 h).

Figure 4C shows the corresponding Tafel plots of FeP NWs/rGO, FeP NPs/rGO, FeP NWs assembles catalysts near the onset of substantial cathodic current, the fitted Tafel plot for the hierarchical nanocomposite of FeP NWs/rGO gives a Tafel slope (*b*) of 58.5 mV dec^−1^, which is much lower than that of FeP NPs/rGO (81.0 mV dec^−1^) and FeP NWs assemblies (101 mV dec^−1^). Based on classical two‐electron reaction model for cathodic HER, this observed Tafel slope of 58.5 mV dec^−1^ suggests a Heyrovsky‐step‐determined Volmer–Heyrovsky mechanism working on the FeP NWs/rGO catalysts.^19^

We further investigated the HER kinetics of the catalysts at the electrode/electrolyte interface by electrochemical impedance spectroscopy (EIS, tested at −0.09 V vs reversible hydrogen electrode (RHE), Figure 4D). The high‐frequency intersection with the *x*‐axis represents ohmic resistance (Rs), mainly arising from the electrolyte and all contact resistances and then used to correct the polarization curves (see Figure S7, Supporting Information). To decouple the ohmic resistance from the polarization resistance, we applied a one‐time constant model of ohmic resistance (Rs) in series with a module (shown in the inset of Figure 4D), where the polarization resistance (Rct) is in parallel with a constant phase element.[[qv: 2c]] The Rct is known to relate to the electrocatalytic kinetics and its lower value corresponds to the faster reaction rate, which can be obtained from the semicircle in the low frequency zone. Compared with that of the FeP NWs assemblies (300 Ω), the much smaller Rct of 109 Ω for FeP NWs/rGO suggests its faster charge transport and faster HER kinetics. Given the above, it is likely this huge discrepancy in charge transfer resistance primarily accounts for the differences in performance. With a similar ohmic resistance and based on their structure analysis, it could possibly be inferred that the vertically oriented FeP array surface facilitates the release of as‐formed gas bubble product because severe adhesion of the gas bubble will block the contact between the catalyst and reactant.

For total electrode activity measurements, the most relevant metric by which to compare catalysts is the potential to reach a current density of interest. As there is no normalization to the amount of catalyst loaded per geometric area, it follows that electrodes with higher catalyst loadings typically have higher electrode activity. In an attempt to resolve this apparent discrepancy, the catalytic properties of the FeP NWs/rGO and other reported FeP HER catalysts toward their loading amount were benchmarked, where the current was normalized by the loading weight of catalyst.[[qv: 1b]],^20^ As seen from Figure 4E, in comparison with those referred FeP catalysts,[[qv: 6b]],[[qv: 7b,c]],[[qv: 10c]] our FeP NWs/rGO exhibited the highest mass activity, suggesting the high HER activity per unit mass. Importantly, the comparison of the mass activity of the referred catalysts at the geometric current density of 10 mA cm^−2^ electrode shows that (inset of Figure 4E), the FeP NWs/rGO catalyst produced a current density as high as 49 mA mg^−1^ catalyst, which was 2.9, 7.3, and 20.4 times greater than that of the FeP nanorods supported on Ti foil[[qv: 10c]] (16.7 mA mg^−1^ catalyst, black bar), FeP nanoarrays supported on CC[[qv: 7b]] (6.7 mA mg^−1^ catalyst, blue bar) and FeP nanoparticles (FeP NPs) supported on CC[[qv: 6b]] (2.4 mA mg^−1^ catalyst, green bar), respectively. Compared to those bulky substrates, GO nanosheets in this work has established itself as an excellent candidate, they lead to strong binding between the rGO nanosheet and the nanostructured FeP nanowires due to the surface oxygen‐containing groups of GO.^21^ On the other hand, the obtained 3D porous‐structured nanoarray electrode could facilitate the mass transport during the hydrogen evolution process and thus enhance the utilization of the catalysts. In particular, it is noted that the catalytic activity of FeP NWs/rGO is also much higher than that of reported FeP NPs supported on GS[[qv: 7c]] (35.7 mA mg^−1^ catalyst, magenta bar). The enhanced activity of our FeP NWs/rGO nanocomposite relative to that of FeP NPs‐GS previously reported may be due in part to the increased accessible surface area produced by the vertically aligned porous nanowires, as well as other intrinsic factors that include better crystallinity and phase purity.

Durability is another important criterion to evaluate the performance of an electrocatalyst. In general, TMPs often suffer from a limited long‐term stability during cycling due to degradation, or corrosion. These factors severely restrict the commercial application of these low‐cost electrode materials. However, the composition with GO is able to mitigate this situation noticeably. As shown in Figure 4F, in contrast to the limited cycling performance of the FeP NWs assemblies toward HER electrocatalysis, the FeP NWs/rGO electrode only shows a slightly decay after the first 500 cycles and retains stable catalytic activity after the 1000th cycles. Furthermore, the FeP NWs/rGO composite catalyst can maintain nearly constant H_2_ (*g*) evolution current at *η* = 130 mV even over 18 h of continuous operation (inset of Figure 4F). This performance is much better than the reported FeP NA/Ti electrode, which showed a large activity drop occurring within 15 h, likely caused by delamination and physical loss of electrocatalyst material.[[qv: 7b]] We believe that the improved stability of FeP NWs/rGO when passing high current densities is contributed collectively by both rGO and FeP nanowire arrays and the unique combined morphology (see Figure S9, Supporting Information). rGO nanosheets are able to undertake some mechanical deformation during the hydrogen‐evolution process of FeP nanowires, which avoided disintegrating the electrode material and was beneficial to a better stability;^22^ while the vertical nanowire arrays were amenable to strain relaxation, which allows them to reduce the disintegration tendency during the alternate processes of bubble accumulation and bubble release.[[qv: 8b]] Moreover, the good contact of the FeP and rGO also played an important role in this process, this is evidenced by the poor stability result of the physically mixed FeP nanorods and rGO (see Figure S10, Supporting Information).

As discussed above, the high catalytic performance of FeP NWs/rGO could reasonably be attributed to three aspects as follows: (1) the direct integration of FeP NWs on the rGO sheets enables strong adhesion and electrical connection between the nanohybrid; (2) the FeP itself possesses a high intrinsic activity and conductivity while the porous hierarchical architecture could be a better structure which favors fast electron transport along the nanowires and in‐time release the as‐formed gas bubble; and (3) the 3D configuration of the vertically oriented FeP NWs on rGO ensure enough open spaces, which allows easy diffusion of electrolyte into all the active sites and consequently more efficient use of the entire electrode. However, many challenges associated with the use of these FeP materials still remain, for example, the improvement of the inherent activity of FeP catalysts. Potential strategies include element doping of the FeP host materials and substrate effects stemming from the interaction of substrate with the host catalyst.^23^

In summary, 3D FeP NWs/rGO nanocomposites were successfully constructed via pseudomorphic transformation of the precursor which consisted of FeO(OH) nanowires vertically aligned on the rGO support. The electrocatalytic properties of 3D FeP NWs/rGO toward HER were also well demonstrated in our studies. Owing to the improved electrode/electrolyte interaction and efficient utilization of the catalysts, 3D FeP NWs/rGO electrocatalyst exhibited enhanced HER performance and better stability than FeP NWs assemblies and FeP NPs/rGO. This study introduces a facile method to construct a hierarchical nanocomposite using 1D and 2D nanocomponents and offers a way for the rational design of new gas evolution materials at the nanoscale level.

## Experimental Section

*Synthesis of Graphene Oxide*: Graphene oxide (GO) was synthesized from natural graphite powder by a modified Hummers' method. Finally, a homogeneous GO dispersion with a concentration of 5 mg mL^−1^ was obtained and used in this work.

*Synthesis of FeO (OH) NWs/rGO*: First, the hierarchical FeO(OH) NWs/GO precursor was synthesized by a simple quasi‐emulsion‐assisted hydrothermal method. In a typical procedure, 140 mg ferrous sulfate (FeSO_4_·7H_2_O, Sigma‐Aldrich) was dispersed in the mixture (38 mL) of water and glycerol with volume ratio of 7:1 by stirring for 15 min. Then 2 mL of aqueous GO solution (containing 10 mg of GO) were added into the above solution and the mixture was ultrasonicated until GO was fully dispersed. Afterward, the above well‐dispersed solution was transferred into a 50 mL Teflon‐lined stainless steel autoclave, followed by heating at 120 °C for 24 h. Then the autoclave was naturally cooled to room temperature and a hydrogel of FeO(OH) NWs/GO was formed and then dialyzed against ultrapure water to remove the residual. After that, the FeO(OH) NWs/GO hydrogel was dried by freeze drying. For comparison, FeO(OH) NWs assemblies were synthesized by following the exactly same procedure above in the absence of graphene oxide. FeP NPs/rGO was synthesized by adding 6 mL of NH_3_·H_2_O (30%) dropwise to the GO suspension (10 mg, 34 mL) containing 140 mg of FeSO_4_·7H_2_O and then, the as‐obtained brown viscous suspension was transferred into a stainless steel vessel and subjected to the hydrothermal reduction at 180 °C for 12 h.

*Synthesis of FeP NWs/rGO*: The hierarchical FeP NWs/GO nanocomposite was finally fabricated by the pseudomorphoic transformation of the FeO(OH) NWs/GO precursors. Briefly, the frozen‐dried FeO(OH) NWs/GO hydrogel and NaH_2_PO_2_ were placed at two separate positions in one closed porcelain crucible with NaH_2_PO_2_ at the upstream side of the furnace. The weight ration of the precursor to NaH_2_PO_2_ is about 1:20. Subsequently, the samples were heated at 350 °C for 2 h with a heating speed of 2 °C min^−1^ in Ar atmosphere. This phosphorization process was also used to produce the FeP NWs assemblies and FeP NPs/rGO.

*Characterization*: All field emission scanning electron microscopy (FESEM) images and EDX spectroscopy spectra were taken on a JEOL JSM 6700F. The TEM images were taken from JEOL JEM 2100F. XRD analysis of different samples was carried out on a X‐ray diffractometer (Bruker AXS D8, Cu K*λ*, *λ* = 1.5406 Å, 40 kV and 20 mA). XPS spectrum was measured on a VG Escalab 250 spectrometer equipped with an Al anode (Al Kα = 1846.6 eV). N_2_ adsorption/desorption isotherm was conduct to analyze the pore structure. The BET surface area was determined using adsorption data in the relative pressure (*P*/*P*_0_) range of 0.05–0.25.

*Electrochemical Measurements*: All electrochemical measurements were conducted on an Autolab PGSTAT302 potentiostat (Eco Chemie, Netherlands) in a three‐electrode cell at room temperature. A graphite rod and a saturated calomel electrode (SCE) were used as counter and reference electrodes, respectively. The working electrode was prepared on a glass carbon (GC) disk as the substrate. Typically, a mixture containing 3.0 mg catalyst, 2.5 mL ethanol and 0.5 mL Nafion solution (0.05 wt%, Gashub) was ultrasonicated for 15 min to obtain a well‐dispersed ink. Then 40 μL of the catalyst ink (containing 40.0 μg of catalyst) was loaded onto a glassy carbon electrode of 5 mm in diameter (loading ≈ 0.204 mg cm^−2^). The presented current density refers to the geometric surface area of the glass carbon electrode. Linear sweep voltammetry with scan rate of 2 mV s^−1^ was conducted in 0.5 m H_2_SO_4_ continuously purged with H_2_ (*g*). The working electrodes were mounted at a rotating disc electrode with a rotating rate of 1000 rpm during the test. In all measurement, the saturated calomel electrode was used as the reference; it was calibrated with respect to RHE. We performed the calibration in the high purity H_2_ saturated electrolyte with a Pt wire as the working electrode. In 0.5 m H_2_SO_4_, *E* (RHE) =*E* (SCE) + 0.259 V. All the potentials reported in our manuscript were referenced to a RHE by adding a value of 0.259 V. AC impedance measurements were carried out in potentiostatic mode at −0.09 V versus RHE from 10^5^ to 0.1 Hz with an AC voltage of 5 mV. The stability tests were examined by continuously cycling the potential between +0.1 and −0.5 V versus RHE at a scan rate of 50 mV s^−1^ while the time‐dependent stability were operated at constant overpotential of 0.13 V.

## Supporting information

As a service to our authors and readers, this journal provides supporting information supplied by the authors. Such materials are peer reviewed and may be re‐organized for online delivery, but are not copy‐edited or typeset. Technical support issues arising from supporting information (other than missing files) should be addressed to the authors.

Supplementary
