# Supplementary material for: Construction of Efficient 3D Gas Evolution Electrocatalyst for Hydrogen Evolution: Porous FeP Nanowire Arrays on Graphene Sheets
Source: Adv Sci (Weinh). 2015 Jun 3;2(8):1500120. doi: 10.1002/advs.201500120 (PMC5032976; doi:10.1002/advs.201500120)
Supplement: Supplementary file 1 — Supplementary [file ADVS-2-0p-s001.pdf]

Copyright WILEY-VCH Verlag GmbH & Co. KGaA, 69469 Weinheim, Germany, 2015.

ADVANCED SCIENCE
Open Access

Supporting Information

for *Adv. Sci.*, DOI: 10.1002/advs.201500120

Construction of Efficient 3D Gas Evolution Electrocatalyst for Hydrogen Evolution: Porous FeP Nanowire Arrays on Graphene Sheets

*Ya Yan, Larissa Thia, Bao Yu Xia, Xiaoming Ge, Zhaolin Liu, Adrian Fisher, and Xin Wang**

Copyright WILEY-VCH Verlag GmbH & Co. KGaA, 69469 Weinheim, Germany, 2013.

Supporting Information

Construction of Efficient 3D Gas Evolution Electrocatalyst for Hydrogen Evolution: Porous FeP Nanowire Arrays on Graphene Sheets

*Ya Yan, Larissa Thia, BaoYu Xia, Xiaoming Ge, Zhaolin Liu, Adrian Fisher, Xin Wang **

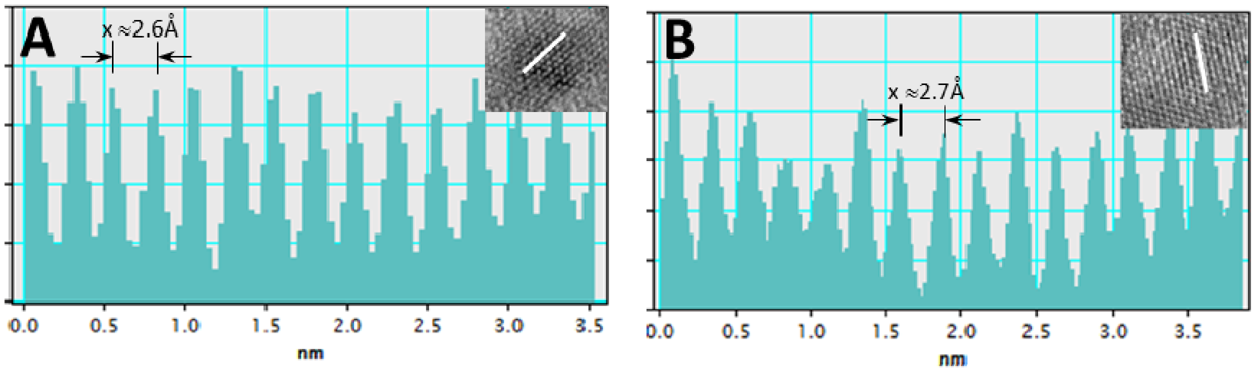

Figure S1. Line scans of FeP NWs/rGO observed in TEM images of Figure 4D. A line scan of the spacing along the (200) (A) and (011) (B) plane show distances of approximately 2.6 Å and 2.7 Å, consistent with XRD measurements and previous HRTEM studies of FeP.

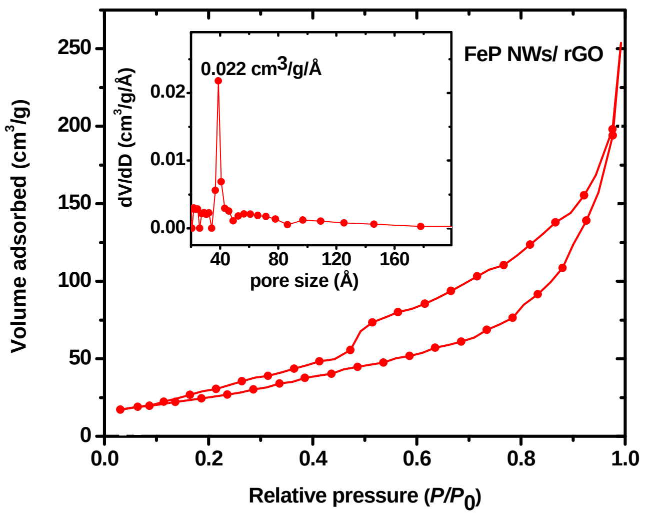

Figure S2. N_2 adsorption−desorption isotherm and (inset) pore size distribution of FeP NWs/rGO nanocomposites.

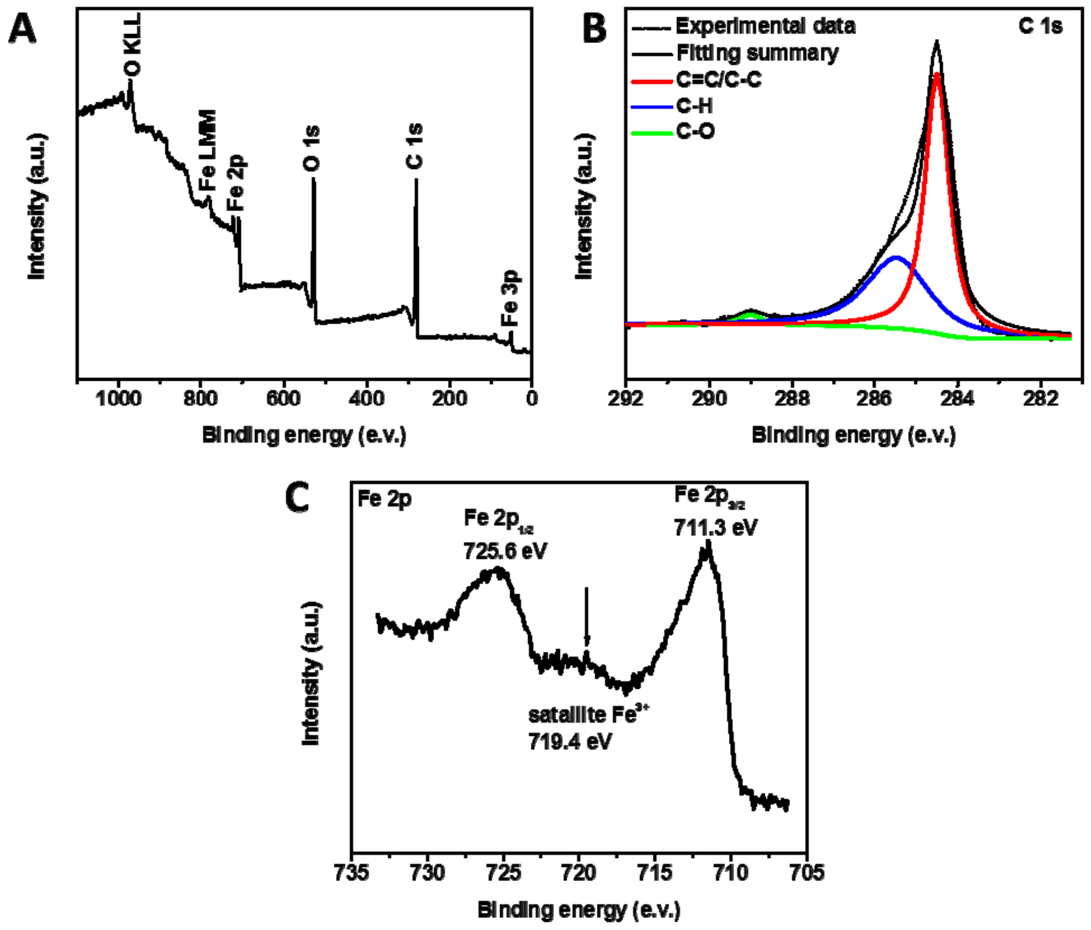

Figure S3. XPS survey scan (A) and high-solution spectra of C 1s (B) and Fe 2p (C), for FeO(OH) NWs/ rGO.

The XPS survey scan of FeO(OH) NWs/ rGO shows three key elements, C, O and Fe, in the sample. The presence of O and Fe elements confirms the successful deposition of FeO(OH) nanowires. The high resolution XPS of C 1s spectrum is deconvoluted into three subpeaks, indicating the existence of three types of carbon. The peaks at 284.5, 285.6, and 288.9 eV are assigned to C=C/C-C, C-O and C=O, respectively. In the Fe 2p spectra, two distinct peaks located at 711.3 and 725.6 eV are observed and correspond Fe $2p_{3/2}$ and Fe $2p_{1/2}$, which can be utilized to qualitatively determine the ionic states of iron. [1] In addition to these two peaks, the occurrence of a satellite peak at about 719.4 eV is consistent with the characterization of Fe^{3+}.[2]

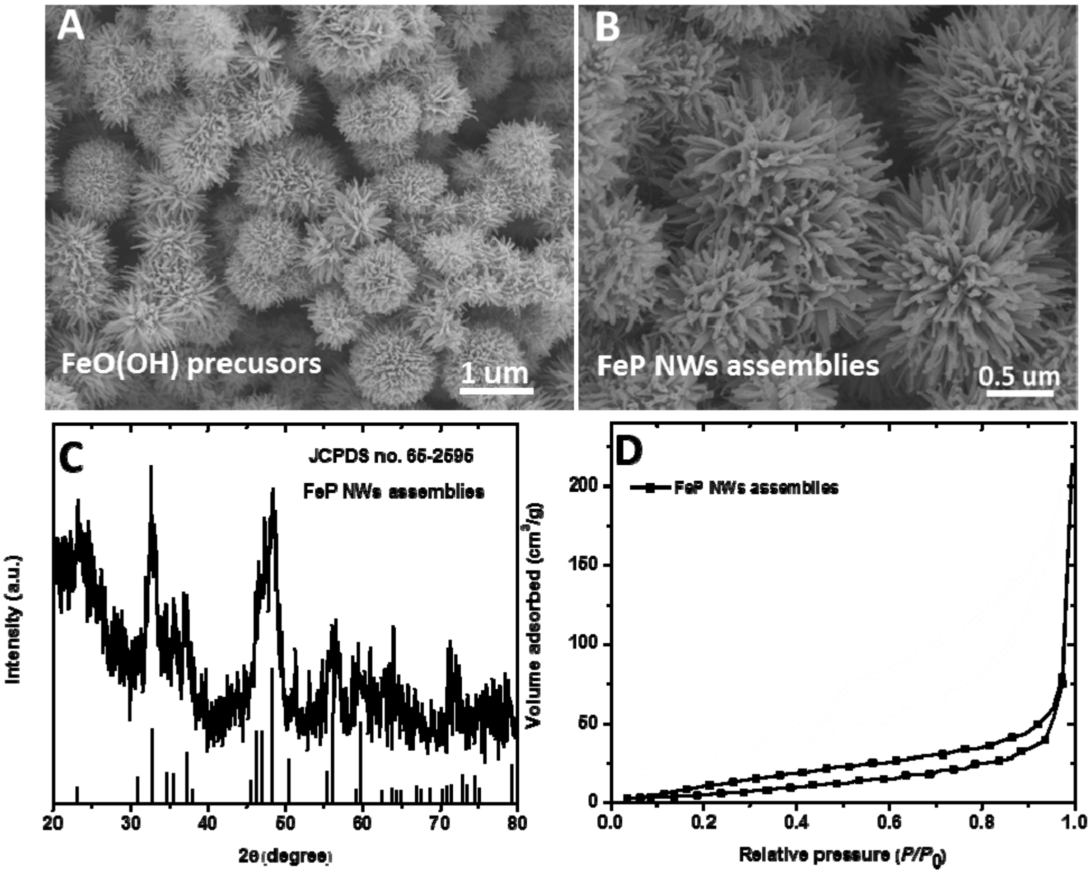

Figure S4. A, B) SEM images of the nanowire assembled FeO(OH) spherical particles and phosphorized product of FeP NWs assemblies. C-D) XRD spectrum and N_2 adsorption−desorption isotherm of FeP NWs assemblies.

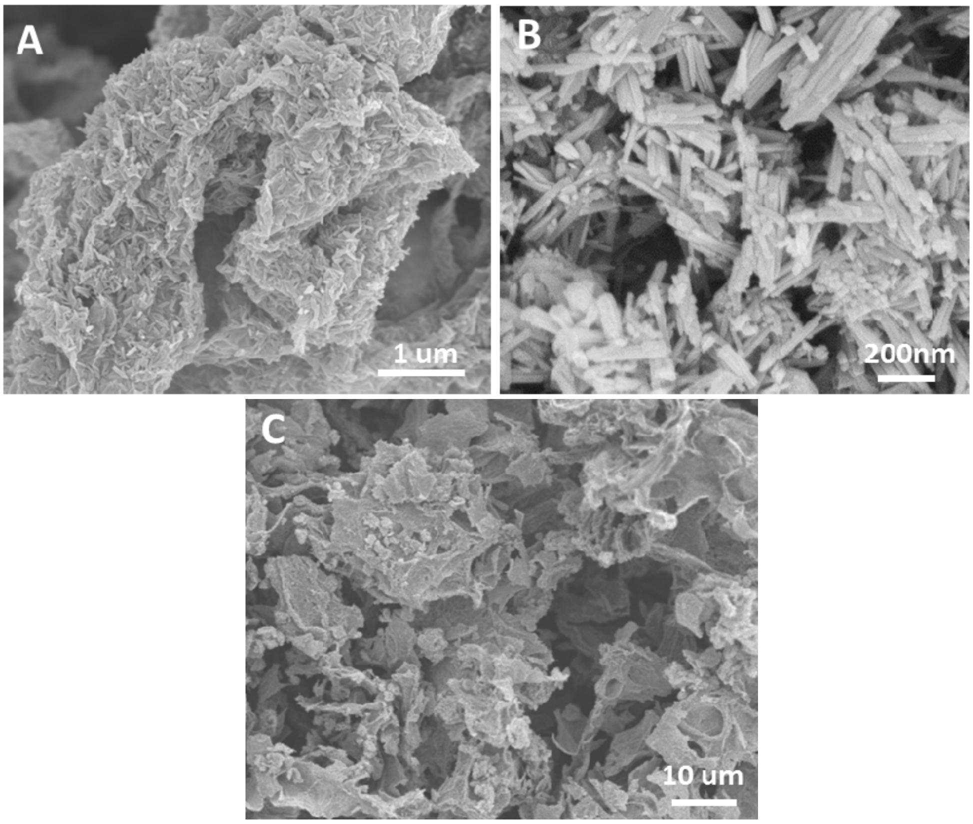

Figure S5. SEM images of the hydrothermal samples obtained without adding of the glycerol (A), the sample without adding both glycerol and GO (B) and the sample dried at 60 ºC for 12 h in an oven(C).

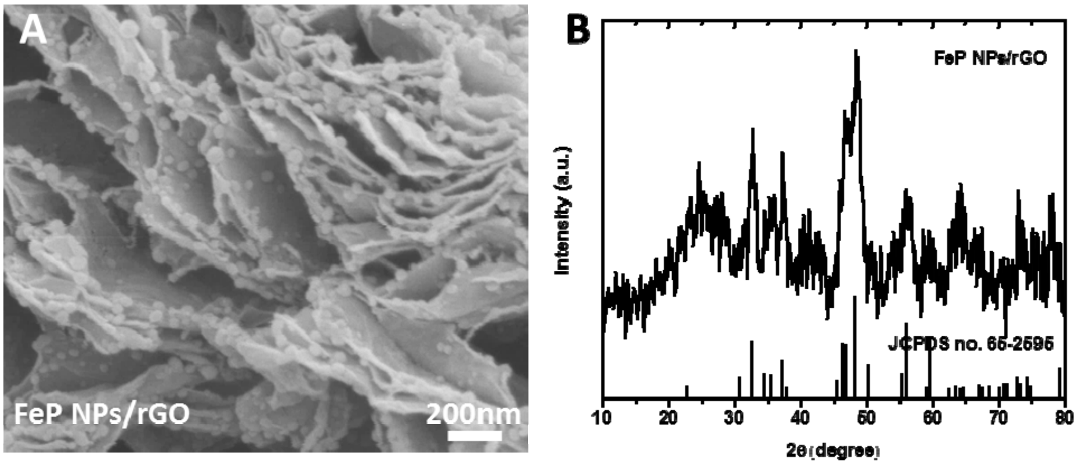

Figure S6. A) SEM image of the FeP NPs/rGO and B) corresponding XRD spectrum.

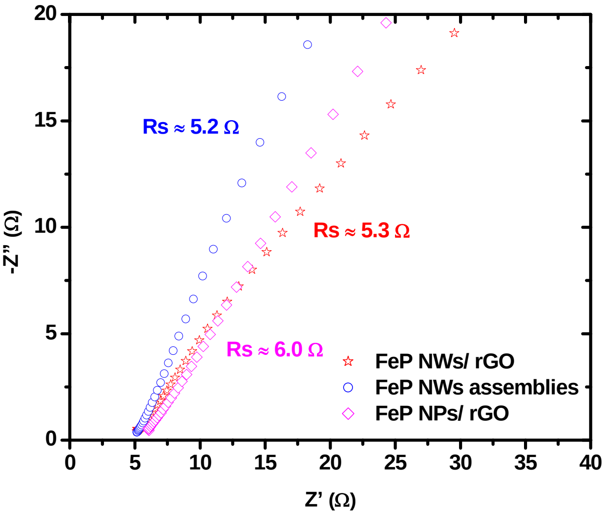

Figure S7. Nyquist plots of the tested samples at high-frequency range. *Z'* is the real impendence and -*Z''* is the imaginary impedance. The intercept of the semicircle on the real axis is assigned to the ohmic series resistance (Rs). *i*R correction to data with the series resistance (Rs) is performed by $\eta_{corr} = \eta - jR$.

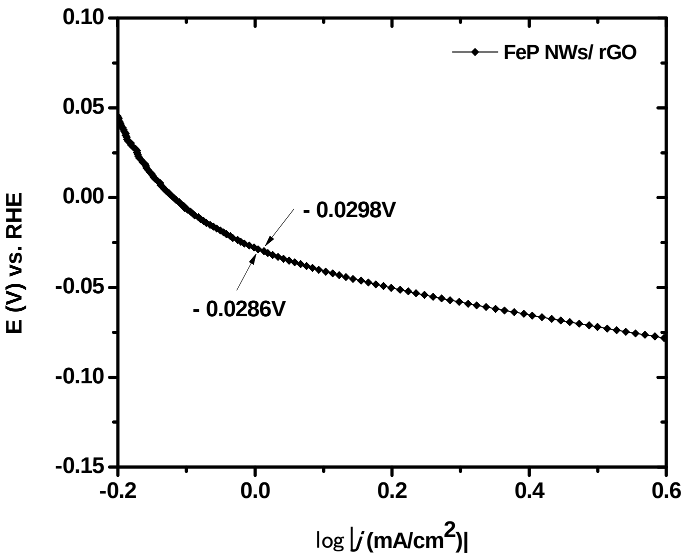

Figure S8. The Tafel plot of the FeP NWs/rGO in the region of low current densities.

The onset potential for HER was read from the semi-log (Tafel) plot. For example, the semi-log plot of FeP NWs/rGO in the region of low current densities as displayed in Figure S5 shows a linear relationship below -0.0298V but starts to deviate above -0.0286V. Therefore, -0.0298V was chosen as the onset potential for FeP NWs/rGO. The same method was applied on determining the overpotential for other samples.

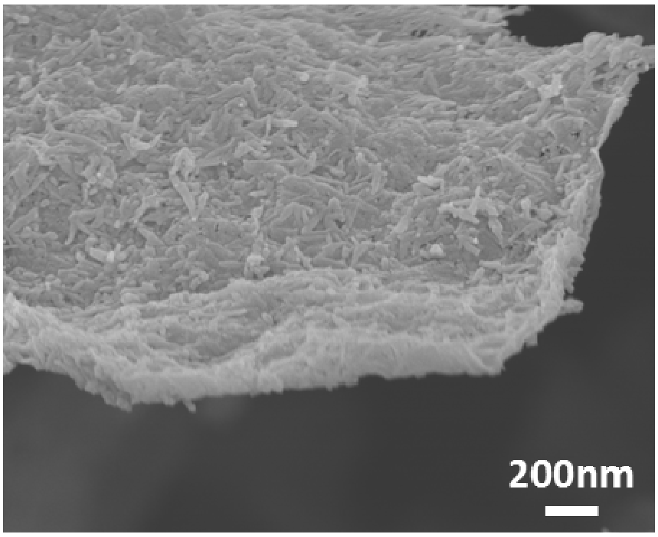

Figure S9. SEM image of the FeP NWs/rGO after 18h stability test under acidic condition.

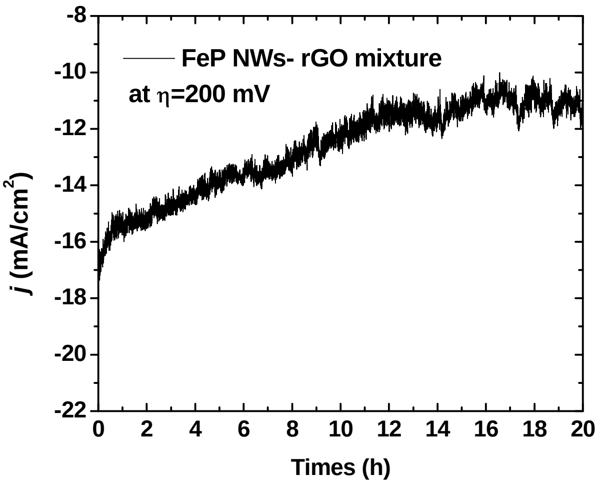

Figure S10. Plot of current density vs. time for FeP NWs and rGO mixture electrode under static η=200 mV for 20 h).

Table S1. Comparison of HER performance in acidic media of FeP NWs/rGO nanocomposite with other non-noble metal electrocatalysts.

Catalyst	Loading amount (mg cm^{-2})	Current density (j, mA cm^{-2})	Overpotential at the corresponding j (mV)	Tafel slope b (mV dec^{-1})	Ref.
MoS_2/Ti	0.12	~30	250	51	Adv. Mater. 2014, 26, 2683
WS_2/rGO	0.40	~5	250	58	Angew. Chem. Int. Ed. 2013, 52, 13751
double-gyroid MoS_2/FTO	0.06	~12	250	50	Nat. Mater., 2012, 11, 963
MoS_2/RGO	0.285	25	180	41	J. Am. Chem. Soc., 2011, 133, 7296
$MoSe_2$/CFP	-	10	250	59.8	Nano Lett. 2013, 13, 3426
WSe_2/CFP	-	~3	250	77.4	
Mo_2C/carbon paste electrode	-	19	250	56	Angew. Chem. Int. Ed. 2012, 54, 12703
MoB/carbon paste electrode	-	17	250	55	
Mo_2C/CNT	2	10	152	65.3	Energy Environ. Sci. 2013, 6, 943
NiMoNx/C	0.25	5	220	35.9	Angew. Chem. Int. Ed., 2012, 51, 6131
MoN nanosheets	0.285	70	300	90	Chem. Sci., 2014,5, 4615
interconnected network of MoP nanoparticles	0.36	~260	250	54	Adv. Mater., 2014, 26, 5702
bulk MoP	0.86	30	180	54	Energy Environ. Sci., 2014, 7, 2624
Ni_2P hollow nanoparticles	1.00	100	180	46	J. Am. Chem. Soc., 2013, 135, 9267
$Ni_{12}P_5$/Ti	1.00	10	137	63	ACS Nano, 2014, 8, 8121
Cu_3P NW/CF	15.2	10	143	67	Angew. Chem. Int. Ed. 2014, 53, 9577
CoP/Ti	2.0	10	90	43	Chem. Mater., 2014, 26, 4326
WP NAs/CC	2.0	10	130	69	ACS Appl. Mater. Interfaces, 2014, 6, 21874
amorphous WP nanoparticles	1.0	10	120	-	Chem. Commun., 2014,50, 11026
FeP nanosheets	0.28	~12	250	67	Chem. Commun., 2013, 49, 6656
FeP NAs/CC	1.5	10	58	45	ACS Catal. 2014, 4, 4065
FeP NAs/Ti	0.6	10	85	60	J. Mater. Chem. A, 2014, 2, 17263
FeP NA/Ti	3.2	10	55	38	Angew. Chem. Int. Ed. 2014, 53, 12855
FeP/CC,	4.2	20	54	32	ACS Appl. Mater. Interfaces, 2014, 6, 20579
FeP-GS	0.28	10	123	50	Chem. Commun., 2014,50, 11554
FeP NWs/rGO	0.204	10	107	58.5	This work

References

[S1] H. Abdel-Samad, P. R. Watson, *Appl. Surf. Sci.* **1997**, *108*, 371.

[S2] T. Yamashita, P. Hayes, *Appl. Surf. Sci.* **2008**, *254*, 2441.
